# Carbon-Based Composite Materials for Electrodes

**DOI:** 10.3390/ma15144908

**Published:** 2022-07-14

**Authors:** Jamballi G. Manjunatha, Bengi Uslu

**Affiliations:** 1 Department of Chemistry, FMKMC College, Mangalore University Constituent College, Madikeri 571201, Karnataka, India; 2 Department of Analytical Chemistry, Faculty of Pharmacy, Ankara University, Tandogan, Ankara 06100, Turkey; buslu@pharmacy.ankara.edu.tr

Carbon-Based Composite Materials for Electrodes is a new open Special Issue of *Materials*, which has the goal of publishing original research and review articles focused on carbon nanotubes, graphene, activated carbon, graphite, pencil graphite, graphene oxide, graphene nanoplatelets, pyrolytic graphite, organic mass derived carbon, fullerenes, diamond, glassy carbon, carbon fibers, and other composites for electrode preparation and its applications.

In the last decade, carbon composites have been playing a significant role in the field of multidisciplinary science and technology. Because the carbon composites are green and economical with brilliant chemical, mechanical, electronic, and surface properties, carbon composite electrodes find many applications in sensing systems, energy storage and management, fuel cell construction, batteries preparation, molecular and ion recognition, electro-synthesis, drug delivery, and more [1,2].

All the possible carbon composites and their applications are welcomed in the current Special Issue entitled “**Carbon-Based Composite Materials for Electrodes**”.
